# Robust prediction of force chains in jammed solids using graph neural networks

**DOI:** 10.1038/s41467-022-31732-3

**Published:** 2022-07-30

**Authors:** Rituparno Mandal, Corneel Casert, Peter Sollich

**Affiliations:** 1 Institute for Theoretical Physics, Georg-August-Universität Göttingen, 37077 Göttingen, Germany; 2 Department of Physics and Astronomy, Ghent University, 9000 Ghent, Belgium; 3 Department of Mathematics, King’s College London, London, WC2R 2LS UK

**Keywords:** Structure of solids and liquids, Statistical physics

## Abstract

Force chains are quasi-linear self-organised structures carrying large stresses and are ubiquitous in jammed amorphous materials like granular materials, foams or even cell assemblies. Predicting where they will form upon deformation is crucial to describe the properties of such materials, but remains an open question. Here we demonstrate that graph neural networks (GNN) can accurately predict the location of force chains in both frictionless and frictional materials from the undeformed structure, without any additional information. The GNN prediction accuracy also proves to be robust to changes in packing fraction, mixture composition, amount of deformation, friction coefficient, system size, and the form of the interaction potential. By analysing the structure of the force chains, we identify the key features that affect prediction accuracy. Our results and methodology will be of interest for granular matter and disordered systems, e.g. in cases where direct force chain visualisation or force measurements are impossible.

## Abstract (web summary)

The emergence of correlated and self-organized linear structures, known as force chains, is relevant for granular materials, foams, emulsions, and extreme active matter. The authors develop a machine learning-based approach to predict force chain formation in jammed disordered solids under deformation.

## Introduction

Force chains are emergent filament-like structures that carry large stresses when a granular material^1–10^, emulsion^11,12^, foam^13^, dense active matter^14^, or assembly of cells^15^ is deformed. Unlike homogeneous simple solids, the stress in such fragile matter propagates inhomogeneously via these force chains^16,17^, which therefore act as a crucial component in describing the mechanical and transport properties of such systems^18–25^. For instance, the knowledge of the force chain network is crucial to understand several key properties of granular materials, such as sound propagation^22^, non-local mechanisms of momentum transfer^26^, or the response to external confining stress^18^. Understanding when force chains will form, how the network that they make up carries the external load and responds to external or internal mechanical deformation ^27–29^, and characterizing the statistical properties of force chains^30–34^ constitute central challenges in ongoing research in granular matter systems. The study of force chains, initially qualitatively^4,6,35^ and later quantitatively^9,28,36–39^, became popular with the introduction of photoelastic beads in granular matter experiments. For example, the visualization of force chains and subsequent analysis have enabled the validation and verification of theoretical models of granular media^6^, and have helped to disentangle the distinguishing features of force chains appearing under different boundary conditions such as shear or uniform compression^7^. A recent study suggests that ants benefit from the force network to remove grains of soil for efficient tunnel excavation^40,41^.

Predicting where a force chain will arise given a deformation, *i.e*. predicting which grains will be part of this emergent structure, is a complex problem if the interactions between the grains are unknown—but tackling it is of vital importance in e.g., material design as the force chains will be a key determinant of a material’s properties^18–25^. While experimental set-ups using the aforementioned photoelastic beads can visualize force chains^7^, it remains impossible in numerous other experiments on granular matter, emulsions, foams, etc. to say where force chains will form without precise knowledge of the interaction between the particles. In this article, we demonstrate an efficient and accurate solution to this open question, by deploying graph neural networks (GNN) to predict the formation of force chains in both frictionless and frictional granular matter.

Machine learning methods have recently shown great potential in the analysis of physical systems, with applications ranging from quantum chemistry to cosmology^42^. In the field of granular matter, softness was introduced as a structural predictor of regions susceptible to rearrangement, based on a classification of human-defined structure functions with a support vector machine^43–45^. Neural networks have recently been used to detect local structure in colloidal systems^46,47^, define a structural order parameter which correlates strongly with dynamical heterogeneities in supercooled liquids^48^, and have helped in uncovering the critical behavior of a Gardner transition in hard-sphere glasses^49^. Neural network-based variational methods have also been introduced to study the large deviations of kinetically constrained models, which are lattice-based systems displaying glassy dynamics^50,51^. Graph neural networks, which operate on the elements of arbitrary graphs and their respective connectivity, have proven successful in predicting quantum-mechanical molecular properties^52^, describing the dynamics of complex physical materials^53^, or providing structural predictors for the long-term dynamics of glassy systems without the need for human-defined features^54^.

**Fig. 1:**
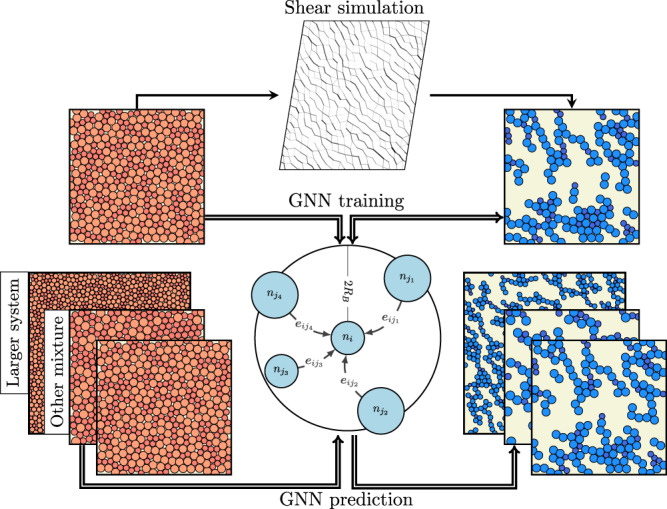
Schematic of our method for predicting the formation of force chains. We first generate data on the formation of force chains via a traditional method, i.e., by shearing a model athermal solid in a simulation setup. We then train a graph neural network to predict the location of force chains in the deformed samples from the initial (undeformed) static structures. The trained graph neural network can then be used to predict the formation of force chains for other initial structures—even when e.g., the system size or particle mixture composition are very different from those used during training.

In this article, we show how a GNN can be trained in a supervised approach to predict the position of force chains that arise when deforming a granular system, given an undeformed static structure (see Fig. 1 for a schematic). For this, we first deform the system using shear deformation (step strain) and identify the particles that become part of force chains using standard methodology (see Methods section). We optimize the GNN on a set of such configurations, training it to predict where the force chains will appear given the initial configurations. We then demonstrate that the trained GNN can generalize remarkably well, allowing it to predict force chains in new undeformed samples. The method is extremely robust: it works exceptionally well in many different scenarios for which the GNN was not explicitly trained, which involve changes in the system size, composition, step strain amplitude, packing fraction, friction coefficient, and even interaction potential—all without requiring any further training. Overall, our method provides accurate and robust predictions of where force chains will appear, without knowledge of inter-grain forces. This method can be potentially applied to numerous experiments on jammed solids, in order to determine the location of force chains even when it is not possible to visualize them directly.

## Results

We first demonstrate that an optimized GNN can predict very accurately where force chains will form for the canonical case of a jammed solid consisting of a binary mixture of harmonic particles. We use *N* particles interacting through a harmonic potential at packing fraction *ϕ* = 1.0, of which *n*_*A*_ particles have radius *R*_*A*_, and *n*_*B*_ particles have radius *R*_*B*_ (as described in Methods).

**Fig. 2:**
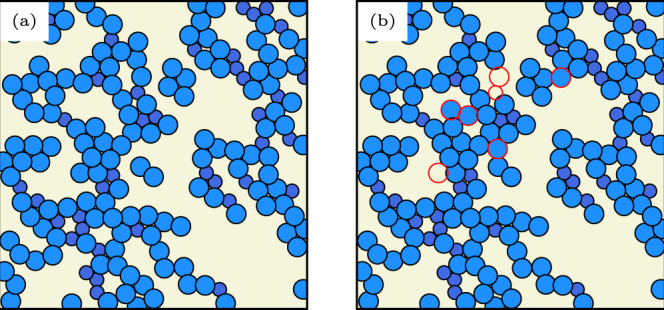
Visualization of force chain predictions for a prototypical athermal solid. **a** Force chains (blue) in an athermal solid with *n*_*A*_ = *n*_*B*_ = 200, obtained through a shear simulation with magnitude of the step strain *γ* = 0.1 at packing fraction *ϕ* = 1.0. **b** Force chains predicted by a graph neural network taking as input the configuration of (**a**) before the deformation was applied. Particles misidentified by the GNN are highlighted in red.

**Fig. 3:**
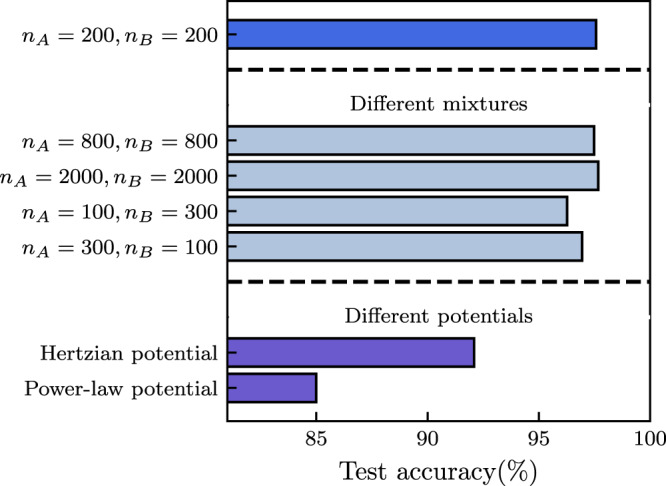
Robustness of force chain prediction accuracy for a variety of physical scenarios. A graph neural network trained on a data set with a harmonic potential and number of particles (top) can also accurately predict force chains for configurations with different system size (number of particles), different ratio of mixture components or with a different interaction potential. All results shown here are obtained at packing fraction *ϕ* = 1.0.

In Fig. 2, we compare the force chains obtained through a numerical shear simulation (Fig. 2a) to those predicted by our trained GNN (Fig. 2b), which receives an undeformed configuration as input. Only a few particles (highlighted in red) are misidentified by the GNN, indicating its very high prediction accuracy, as described in Fig. 3. In the following sections, we describe the most useful aspects of the GNN predictor for the prediction of force chains, namely scalability and robustness.

### Scalability

As each graph-convolutional operation in our GNN only depends on a particle’s local neighborhood (Eq. (4) from Methods), the GNN is not explicitly dependent on the number of nodes in the graph. This allows us to apply the GNN on systems containing a much larger number of particles than it was initially trained on, as long as the relevant physical length-scales stay of the same order when increasing system size. Doing so allows for massive numerical acceleration in the study of large-scale systems, as the largest computational cost for training the GNN lies in the generation of a large enough training data set and in the optimization of the network’s hyperparameters—both of which are much more expensive when training on large systems. We demonstrate the success of this strategy in Fig. 3, where we train a GNN on data acquired for small system size (*N* = 400) and then apply it to much larger systems (shown up to *N* = 4000) without a decrease in force chain prediction accuracy. This is important in the context of predicting force chains in experiments: once trained, evaluating our prediction method only demands a cost that increases linearly with system size so that one can easily make predictions on the much larger systems one has to deal with in a typical experiment. More results on scalability near jamming are provided in the SI.

### Robustness

**Fig. 4:**
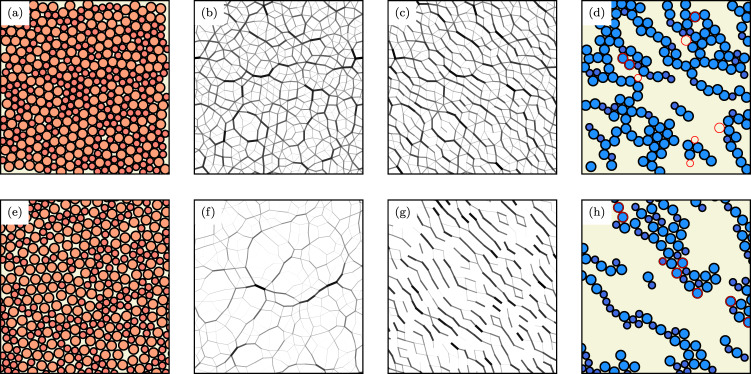
Visualization of force network evolution under deformation and corresponding neural network prediction accuracy. **a** Example configuration before deformation with *n*_*A*_ = *n*_*B*_ = 200 particles interacting through a harmonic potential (as given in Eq. (1)) at packing fraction *ϕ* = 1.0. **b** Corresponding force network. **c** Force network after deformation with step strain of amplitude *γ* = 0.1. **d** Force chains of the configuration in (**c**). Differences between simulation and GNN prediction on the basis of (**a**) are highlighted in red. **e**–**h** Same as in (**a**–**d**) but now for a configuration at a packing fraction (*ϕ* = 0.845) close to jamming.

We first observe that a GNN can predict force chains very accurately when either trained on a data set generated at high (*ϕ* = 1.0) or low packing fractions (*ϕ* = 0.845, close to jamming); Fig. 4a, e show sample configurations. Two separate GNNs were trained on data obtained at the two packing fractions. Fig. 4d, h show their predictions (on previously unseen, undeformed configurations) of force chains, which match remarkably well with the direct simulations at both high and low packing fraction. This consistent accuracy is achieved even though the force networks at the different packing fractions have a very distinct structure, as can be seen from Fig. 4b, f, c, g showing the configurations before and after deformation, respectively.

**Fig. 5:**
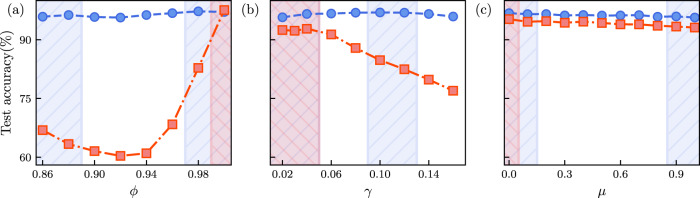
Interpolation and extrapolation performed by the graph neural network on changing control parameters. **a** Force chain prediction accuracy for systems at *γ* = 0.1 and with different packing fractions, ranging between *ϕ* = 0.85 and *ϕ* = 1.0, with a GNN conditioned on *ϕ*. We show results obtained with a GNN trained on a data set obtained at *ϕ* = 1.0 (red), and a GNN trained on data of samples obtained at *ϕ* ≤ 0.88 and *ϕ* ≥ 0.98 (blue). The latter GNN provides highly accurate predictions in the interpolation regime 0.9 ≤ *ϕ* ≤ 0.96, even though it was never trained on these values of the packing fraction. **b** Same as in (**a**) but now for GNNs conditioned on the step strain amplitude *γ*. A GNN trained on *γ* ≤ 0.04 and 0.10 ≤ *γ* ≤ 0.12 (blue) successfully interpolates between these deformation magnitudes; it also extrapolates reliably to larger values of *γ*. A GNN trained only on *γ* ≤ 0.04 (red) fails to do this accurately. **c** Same as in (**a**) and (**b**), but now for a GNN conditioned on the friction coefficient *μ*. Both the GNN trained only on *μ* = 0.0 (red) and the GNN trained on *μ* = {0.0, 0.1, 0.9, 1.0} (blue) provide accurate results across all values of *μ*. The small systematic difference in testing accuracy can be explained by the latter GNN having access to four times as many training examples overall.

A network trained on a fixed, single value of the packing fraction *ϕ* provides inaccurate predictions when applied to configurations obtained at other packing fractions (see Fig. 5a). If, however, we provide the GNN with information about the packing fraction during training, it can correctly predict force chain formation over a wide range of values for the packing fraction, even for values not included in the training data. Likewise, we can provide the neural network with the magnitude of the step strain *γ*, and apply it to correctly predict force chains when varying the value of *γ* for a given configuration. Again, this works even for values of *γ* for which the GNN did not observe any data during optimization: in Fig. 5b we demonstrate that the GNN produces highly accurate predictions both when interpolating between values of *γ* included in the training set, and when extrapolating towards higher values of *γ*. The trained GNN is also robust to changes in the composition of the binary mixture. To demonstrate this, we generate samples with a very different composition by changing the ratio of *n*_*A*_:*n*_*B*_, while keeping the packing fraction fixed. This is demonstrated in Fig. 3 for the cases of *n*_*A*_:*n*_*B*_ = 3:1, and *n*_*A*_:*n*_*B*_ = 1:3, with a GNN that was originally trained on data with *n*_*A*_:*n*_*B*_ = 1:1. The predictions are remarkably good for both cases, with only a minimal loss of accuracy when compared to the original mixture composition.

The fact that a GNN can provide predictions without any significant loss in accuracy in these three different generalization scenarios is highly advantageous, as it implies that large amounts of data only need to be obtained (either through experiment or simulation) at a few model parameter combinations, thus greatly reducing the cost for predicting where force chains will form in a generic experiment.

Next, we ask our GNN to predict the force chains in a jammed solid with different pairwise interactions—either through a Hertzian potential or a power-law potential (see Methods). We find that even in these cases, the GNN retains most of its accuracy (Fig. 3), with a larger performance reduction for the power-law potential. This robustness to the nature of the interaction potential is extremely important for the applicability of our method in an experimental context, where the particle interactions might not be straightforward to estimate.

We have further extended our study to frictional jammed granular solids^55,56^ (see details of the frictional simulation in the Supplementary Information). We first observe that our prediction using the GNN works equally well in the case of frictional particles with friction coefficient *μ* = 1.0, with a similar prediction accuracy of ~96% (see Fig. 6 in the Supplementary Information). Next, we train a GNN on a data set consisting of configurations obtained for *μ* = {0.0, 0.1, 0.9, 1.0}, where the GNN is conditioned on the value of *μ* by including it as a feature for each node in the graph (we hence train on all these values of *μ* simultaneously). Once training is completed, we use the set of weights that achieved the lowest loss on a validation data set consisting of configurations obtained at the same values of *μ* = {0.0, 0.1, 0.9, 1.0} as used during training. We then apply the network with these weights to predict the force chains for unseen test configurations with *μ* = {0.0, 0.1, 0.2, …, 0.8, 0.9, 1.0}. Note that the GNN was not trained on frictional coefficients in the range 0.2 ≤ *μ* ≤ 0.8, so the GNN has to interpolate between its learned classification at high and low values of *μ*. We observe that this interpolation can again be achieved with extremely high accuracy of ~96%, as plotted in Fig. 5c. As an additional check of robustness, we also generated strained packings of frictional particles using a finite deformation rate, with particle configurations relaxing as they are strained in contrast to the previous analysis where we implemented only affine strain. Though slightly less accurate than for the case of step strain, the GNN is still able to classify particles as being part of a force chain with an accuracy of approximately 90%.

**Fig. 6:**
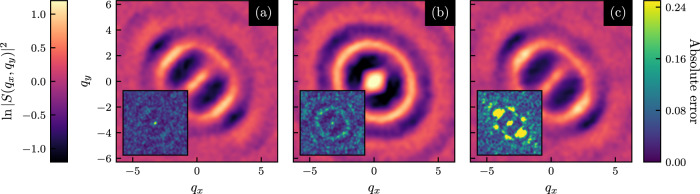
Structure factors of force chains predicted by the graph neural network. **a** Structure factor of force chains predicted by a GNN at *ϕ* = 0.86; the GNN was conditioned on the packing fraction *ϕ*, and trained on data for *ϕ* = {0.86, 0.88, 0.90}. **b** Same as in (a), but now predictions are made at *ϕ* = 1.0, for which the GNN was not trained. **c** Structure factor of force chains predicted at *γ* = 0.16 by a GNN conditioned on *γ* and trained on *γ* = {0.02, 0.03, 0.04}. Inset (in all three cases) shows an absolute error of the structure factor w.r.t. the structure factor of the exact force chains; a distinct pattern can be observed in the inset of (**b**) and (**c**).

Analysis of structure factors of the force chains predicted by the GNNs reveals that the structure factor is quite different at different packing fractions: at *ϕ* = 0.86, it is strongly anisotropic, while at *ϕ* = 1.0 it is nearly isotropic due to the presence of more branched chains; see SI Fig. 7. For two different frictional cases (see SI Fig. 8), on the other hand, very similar structures result. This is consistent with, and rationalizes, the fact that training only on data with *μ* = 0.0 is highly effective for predicting force chains at larger values of *μ*, whereas performing an extrapolation toward higher values of *ϕ* while only training on data for *ϕ* = 0.86 is not (for more details see Supplementary Information). It also underscores the relevance of the GNN in establishing the fact that at high enough packing fractions, friction does not play a significant role and demonstrates that the GNN prediction in an unknown system is able to provide important information about the force chains under different, physically relevant conditions. To explore this matter further, we show the structure factor of the force chains predicted by GNNs in different scenarios in Fig. 6, along with the absolute prediction errors. This structure factor is highly accurate when the GNN is tasked with predicting force chains for conditions on which it is trained (Fig. 6a), or where it can interpolate. However, when the GNN is asked to extrapolate far from its training regime (Fig. 6b, c) strong systematic bias is present in this structure factor. This reveals structural properties the GNN misses out on when performing its extrapolation, such as branching events. We provide more details on the presence of this systematic bias in the extrapolation regime of GNNs in the Supplementary Information.

## Discussion

In this article, we made use of graph neural networks (GNN) to accurately predict where force chains will arise upon the deformation of jammed disordered solids (both frictional and frictionless). Our network is trained on data obtained through direct shear simulations and exhibits a very high generalization ability to unseen configurations. Crucially, the optimized GNN is robust to changes in a variety of model parameters, such as the packing fraction, system size, magnitude of deformation, friction coefficient, and interaction potential: it produces very accurate predictions for such cases, without having ever observed them during its training. Although we have used data obtained through numerical simulations to train our GNN, the methodology would be identical for experimental data, where it is not always possible to measure inter-particle forces. We have also analysed the structure factor of the predicted force chains in different physical scenarios, and discussed the implication on the performance of GNN while doing extrapolation. We have verified (see Supplementary Information for details) that the predictions of our GNN are not correlated with the local ${D}_{\min }^{2}$, an indicator of plastic rearrangements. This can in turn be predicted using softness, which is a machine learning feature that has recently been used extensively^43–45^. Overall, this indicates that our GNN is picking up on novel features in the local structure of such disordered solids.

Our study will open new possibilities in experiments on such disordered solids (granular matter, emulsions, foams, etc.) where direct visualization of force chains is not possible, allowing for more in-depth analysis of structural properties as quantified by force chains. Even though for the demonstration of the success of our method we have used data generated by simulations of two-dimensional grains it is straightforward to extend the method to three-dimensional configurations where visualization of force chains in experiments is a very challenging task^11,57,58^.

## Methods

### Model and simulation

We first train our GNN on configurations of a frictionless^59–63^ athermal soft binary sphere mixture with harmonic interactions^64–66^ as a model athermal solid. The pair-interaction potential between two particles *i* and *j* is completely repulsive, and of the form

1 $$V({r}_{ij})=\frac{1}{2}G{R}^{3}{\left(1-\frac{{r}_{ij}}{{R}_{i}+{R}_{j}}\right)}^{2}$$

when particles overlap and zero otherwise. Here *R*_*i*_ and *R*_*j*_ are the radii of the *i*-th and *j*-th particle respectively, *r*_*ij*_ is the distance between them, and *R* is a characteristic particle radius parameter. All the lengths in the problem are described in units of *R* and energies in units of *GR*^3^. For generating the initial jammed structure we employed overdamped athermal dynamics with friction coefficient ζ. This, along with the choices for *R* and *G*, sets the time unit as ζ/(*GR*). Our initial simulations (for training data generation) are performed using a binary mixture of *n*_*A*_ = *n*_*B*_ = 200 particles with *R*_*A*_ = *R*, *R*_*B*_ = 1.4*R* in a two-dimensional box of linear size *L* and with periodic boundary conditions. We have also used a Hertzian potential of the form

2 $$V({r}_{ij})=\frac{2}{5}G{R}^{3}{\left(1-\frac{{r}_{ij}}{({R}_{i}+{R}_{j})}\right)}^{5/2},$$

and power-law potential

3 $$V({r}_{ij})=\epsilon {\left(\frac{{R}_{i}+{R}_{j}}{{r}_{ij}}\right)}^{10}$$

where we choose the energy scale for the Hertzian potential as before, and as *ϵ* for the power-law potential. For these training data generation runs, the simulations are performed at a fixed packing fraction *ϕ*, which is defined as $\phi =({n}_{A}\pi {R}_{A}^{2}+{n}_{B}\pi {R}_{B}^{2})/{L}^{2}$. We always start with a force-free configuration under athermal conditions, and then deform the system by applying a step shear strain of magnitude *γ*. Lees-Edwards periodic boundary conditions are used to implement the step strain. The simulation methodology for the frictional jammed systems, which are discussed in the section on robustness, is described in the Supplementary Information.

### Identification of force chains

To quantitatively detect force chains we follow the approach detailed in^67,68^, where force chains are defined as quasi-linear structures formed of particles that carry above-average load, i.e., compressive stress. To identify the particles within the force chains, we first calculate the stress tensor ${\hat{\sigma }}_{\alpha \beta }=\mathop{\sum }\nolimits_{i = 1}^{{N}_{nb}}{f}_{\alpha }^{i}{r}_{\beta }^{i}$ for each particle in the instantaneously sheared configuration, where *N*_*nb*_ is the number of neighboring particles exerting a force on the central particle, and ${f}_{\alpha }^{i}$ and ${r}_{\beta }^{i}$ are the components of the force and the radius vector connecting the centers of the two interacting particles, respectively. The largest eigenvalue of ${\hat{\sigma }}_{\alpha \beta }$ is the magnitude of the particle load vector, while its orientation is given by the corresponding eigenvector. Neighboring particles whose load vectors align within an angle of 45^∘^ (in Fig. 4 of the SI and the associated discussion we show that the GNN’s accuracy does not depend on the choice of this angle.) and have above-average (arithmetic mean) magnitude are assigned as part of a force chain^67,68^. In a completely analogous fashion, one could train neural networks on force chains identified using other approaches, *e.g*. based on community detection^69,70^ or topological properties of the force network^71^. Note that these detection techniques^67–71^ allow for the analysis of the force network, but do not provide any predictive power on how it will change upon deforming a system.

### Training of GNN and prediction of force chains

To predict the location of force chains after a deformation with graph neural networks, we first transform a given initial configuration into a graph by drawing edges between particles that are within a fixed cutoff distance (here set to 2*R*_*B*_). Each node of this graph has the corresponding particle radius as feature *n*_0_. When conditioning our graph neural network on a global property such as the magnitude of the deformation *γ* or packing fraction *ϕ*, we include this property as an additional (uniform) node feature. The features assigned to each edge of the graph *e*_*ij*_ consist of the distance between the two particles connected by the edge and the unit vector in the direction of their relative distance. Importantly, we do not need to include any knowledge of the contact forces between the particles, which are typically difficult to measure^37,72^.

In order to predict which particles will become part of force chains after deforming the initial configuration, we apply a graph neural network to this graph. Such a GNN consists of *N*_*l*_ layers, where in every layer *l* the node features corresponding to each particle *i* are updated according to the features of the particles in their neighborhood ${{{{{{{\mathcal{N}}}}}}}}(i)$ and the features of the edges that connect them (see schematic in Fig. 1). Specifically,

4 $${({n}_{l})}_{i}={({n}_{l-1})}_{i}+\mathop{\sum}\limits_{j\in {{{{{{{\mathcal{N}}}}}}}}(i)}{f}_{{{{{{{{\mathcal{W}}}}}}}}}^{l}\left[{({n}_{l-1})}_{i},{({n}_{l-1})}_{j},{e}_{ij}\right],$$

where ${f}_{{{{{{{{\mathcal{W}}}}}}}}}^{l}$ is a parameterized nonlinear function (neural network) that calculates new node features for each particle. Importantly, we use the same function ${f}_{{{{{{{{\mathcal{W}}}}}}}}}^{l}$ for each particle, which allows us to apply the GNN to systems with an arbitrary number of particles.

For our force chain prediction, we pass the features ${n}_{{N}_{l}}$ of each node in the final layer through a fully-connected neural network with one output feature and a sigmoid activation function. This final result is, for each particle, the probability $\hat{p}$ of being part of a force chain after deformation, as assigned by the neural network.

During training we optimize the weights of the GNN such as to minimize the cross-entropy^73^

5 $${{{{{{{\mathcal{H}}}}}}}}(p,\hat{p})=-\frac{1}{NM}\mathop{\sum }\limits_{i=1}^{NM}\left[{p}_{i}\log ({\hat{p}}_{i})+(1-{p}_{i})\log (1-{\hat{p}}_{i})\right],$$

where *M* is the number of configurations in the training set and *N* is the number of particles in each configuration, *p* = 1 if a particle is part of a force chain in a particular training configuration, and *p* = 0 otherwise. We use the Adam optimizer^74^ to minimize this loss function on a training set, and choose the model hyperparameters as those that perform best on a validation set consisting of configurations not included in the training set. We then evaluate our network on an independent test set. The optimized hyperparameter values and more details on the neural network architecture and training can be found in the SI.

## Supplementary information

Supplementary Information

## Data Availability

A representative data set generated for the results in the main text and in the supplementary information has been deposited in the Göttingen Research Online database under accession code 10.25625/TCFCVI. The complete data are available from the authors upon reasonable request.

## Code availability

A representative code used to do the ML analysis has been deposited in the Göttingen Research Online database under accession code 10.25625/TCFCVI. The complete code is available from the authors upon reasonable request.
