# Supplementary material for: Robust prediction of force chains in jammed solids using graph neural networks
Source: Nat Commun. 2022 Jul 30;13:4424. doi: 10.1038/s41467-022-31732-3 (PMC9338954; doi:10.1038/s41467-022-31732-3)
Supplement: Supplementary file 1 — Supplementary Information [file 41467_2022_31732_MOESM1_ESM.pdf]

Supplemental Information for "Robust Prediction of Force Chains in Jammed Solids using Graph Neural Networks"

MODEL ARCHITECTURE AND TRAINING DETAILS

Given an undeformed configuration, the first step in our force-chain prediction routine is to create a graph by identifying the particles as nodes and drawing edges between nodes that are separated by a maximum distance of $2R_B$. We assign a feature vector e_0 to each edge, which contains the distance and relative orientation of the particles it connects; and a feature vector n_0 to each node, which contains the particle radius and any global features being conditioned on, such as the packing fraction or the amplitude of the step strain deformation. We then embed the node features n_0 and edge features e_0 of the initial graph in a higher-dimensional space using a parameterized linear transformation

$$n_0' = W^{(n)} n_0 + b_n, \quad (1)$$
$$e_0' = W^{(e)} e_0 + b_e. \quad (2)$$

Here, $W^{(n)}$ and $W^{(e)}$ are weight matrices of dimension $(d_h \times |n_0|)$ and $(d_h \times |e_0|)$ respectively and b_n and b_e are d_h-dimensional bias vectors; d_h is a hyperparameter called the hidden dimension. We then pass these features through N_l layers of a residual graph-convolutional network. In each of the layers l, we calculate new node features n_l' for each particle i from the features of its neighbouring particles $\mathcal{N}(i)$ and the connecting edges, by applying a linear transformation followed by a non-linear activation function. The features n_l' are then added to the original features n_{l-1}', which allows for more stable training. Explicitly, we use the graph-convolutional operator [1]

$$(n_l')_i = (n_{l-1}')_i + \sum_{j \in \mathcal{N}(i)} \sigma(W_l^f (z_{l-1})_{i,j} + b_l^f) \odot g(W_l^s (z_{l-1})_{i,j} + b_l^s). \quad (3)$$

Here, $(z_{l-1})_{i,j}$ is the vector obtained by concatenating the node features $(n_{l-1}')_i$ and $(n_{l-1}')_j$ with the edge features $(e_0')_{i,j}$, $\sigma(x) \equiv 1/(1+e^{-x})$ and $g(x) \equiv \ln(1+e^x)$ are a sigmoid and softplus function respectively, and $\odot$ represents element-wise multiplication. W_l^f and W_l^s are trainable weight matrices of size $d_h \times 3d_h$ acting on the node and edge features, while b_l^f and b_l^s are d_h-dimensional bias vectors. Note that the weights and biases are identical for each node. This type of graph-convolution provided the most accurate predictions in our numerical experiments; other graph convolutions we tested resulted in a lower accuracy, including the graph-convolutions used in [2–4] (implemented using [5]).

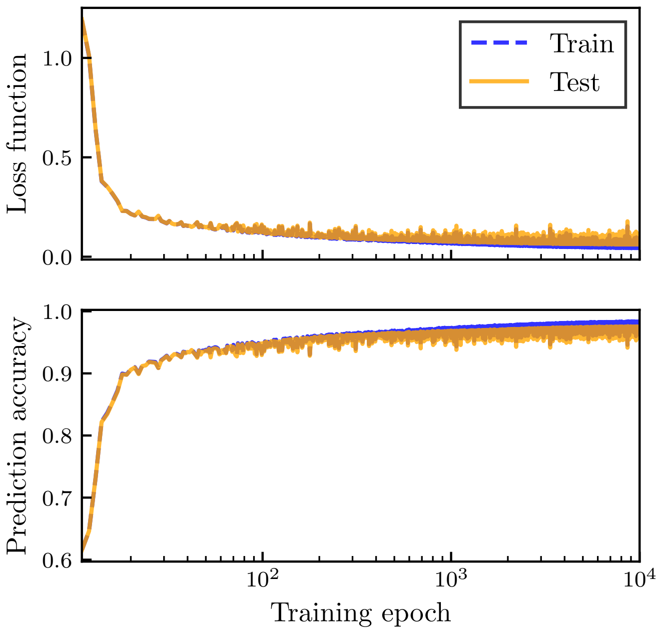

Supplementary Figure 1. Loss function (cross entropy) and prediction accuracy during training, evaluated on a training set consisting of 1024 configurations and a testing set consisting of 128 configurations.

During training we optimize the weights of the GNN such as to minimize the cross-entropy [6]

$$\mathcal{H}(p,\hat{p}) = -\frac{1}{NM}\sum_{i=1}^{NM}\left[p_i \log(\hat{p}_i) + (1-p_i)\log(1-\hat{p}_i)\right], \quad (4)$$

where M is the number of configurations in the training set and N is the number of particles in each configuration, $p = 1$ if a particle is part of a force chain in a particular training configuration, and $p = 0$ otherwise.The weight updates are performed using the Adam optimizer [7]. In Fig. 1, we show how the loss decays and the accuracy increases (both on a training and testing set) during a typical training routine.

In order to choose the model and training hyperparameters (*i.e.* the learning rate λ for the Adam optimizer, the hidden dimension d_h and the number of layers N_l; we used a fixed batch size of 64 configurations) that provide the most accurate predictions, we optimized a GNN for many combinations of these hyperparameters on a data set of 1024 configurations of $n_A = n_B = 200$ particles at a packing fraction of $\phi = 1.0$. We then evaluated the loss function on an independent validation set consisting of

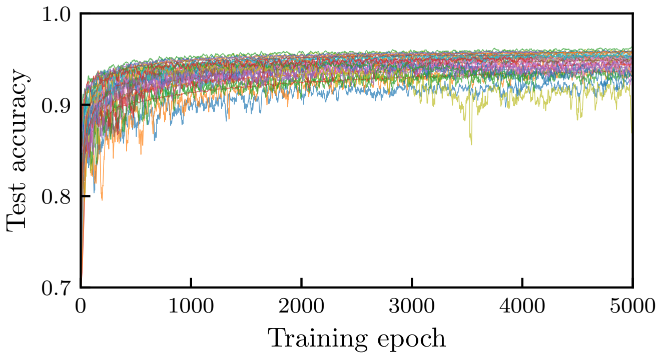

Supplementary Figure 2. Evolution of the prediction accuracy on a validation set during training of the GNN. Each line represents a different combination of hyperparameter values. We considered values of d_h between 16 and 128, of N_l between 2 and 12, and of λ between 0.0001 and 0.1.

128 configurations, and chose the hyperparameters that resulted in the lowest loss: $d_h = 64,\ N_l = 8,\ \lambda = 0.001$. All results shown in the manuscript were obtained with this set of hyperparameters. We used 1024 training configurations for each set of different values of the control parameters (*i.e.* combination of packing fraction ϕ and amplitude of step strain γ), while the reported test accuracy is evaluated on 128 independent configurations.

We note here that our method is quite robust against changing the values of the hyperparameters mentioned above, and GNNs containing far fewer hyperparameters did not result in significantly worse prediction accuracy. In Fig. 2, we visualize how the accuracy on the validation set increases during training for many different hyperparameter combinations; each tested combination resulted in an accuracy of at least 90%.

One concern was that the influence of long-range correlations near jamming (*i.e.* $\phi = 0.845$) might negatively affect our ability to obtain accurate predictions for systems larger than those trained on, or require excessive network depth to incorporate these long-range correlations. In Fig. 3, we demonstrate that this is not the case: even very shallow GNNs consisting of just two layers can predict force chains in systems containing ten times as many particles as seen during training, with an accuracy of 93%.

ROBUSTNESS WITH RESPECT TO FORCE CHAIN IDENTIFICATION

Following Refs. [8, 9], we have assigned neighbouring particles whose load vectors align within an angle of $\alpha = 45°$ and have above-average (arithmetic mean) magnitude as part of a force chain. In Fig. 4, we show that the value of α chosen to identify the force chain has no influence on the GNN classification accuracy. Here, for each value of α ranging between 30° and 60°, we trained a new GNN on a data set consisting of the same configurations but with labels depending on the value of α used to extract the force chains. This result once again showcases the robustness of this method, and is relevant for applications in experimental settings where different values of α might be used to define force chains.

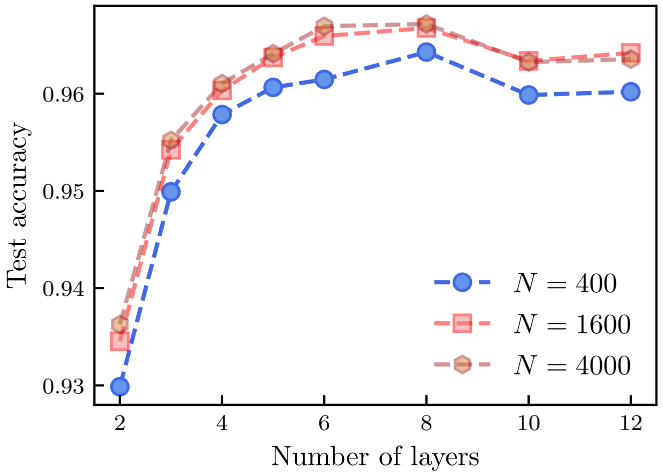

Supplementary Figure 3. Test accuracy for systems at $\phi = 0.845$ with number of particles between 400 and 4000, as a function of the number of layers of the GNN.

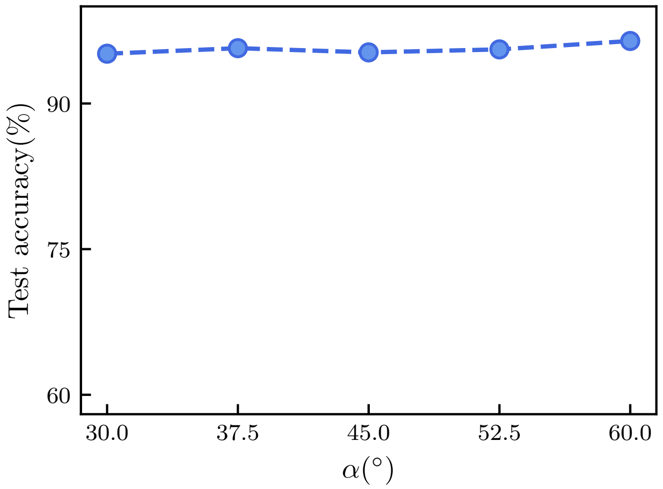

Supplementary Figure 4. Test accuracy of GNNs trained on force chains detected with different values of α, the threshold angle between load vectors which has been used to decide whether the particle in question is part of the force chain or not.

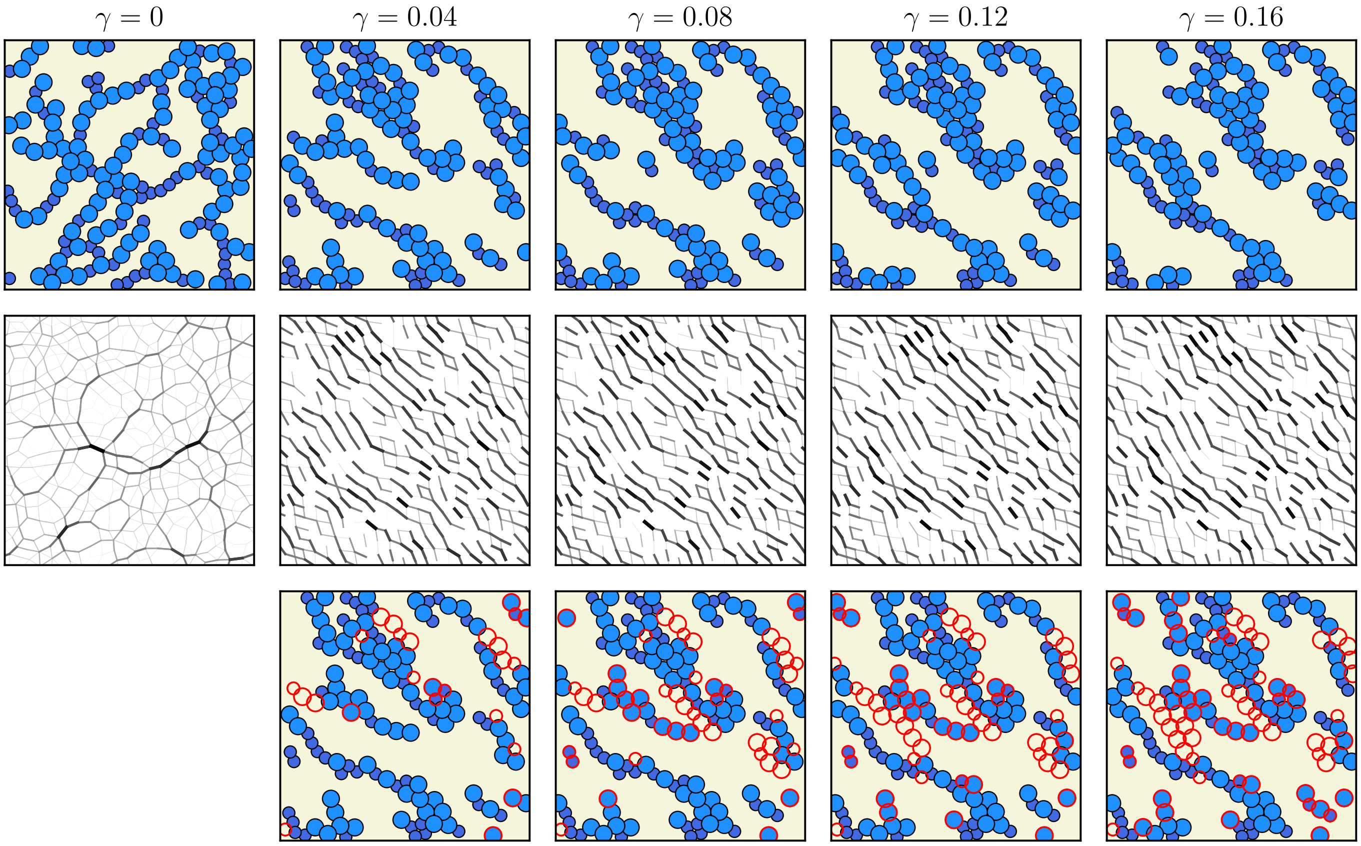

Supplementary Figure 5. Top row: Force chains for a configuration obtained with different values of γ. Middle row: the corresponding force network. Bottom row: In blue, we show the predictions made by a GNN only trained to predict chains for $\gamma = 0.02$. The red circles indicate the difference between the actual force chains and those predicted by the GNN, indicating a decrease in overlap as γ increases.

DEPENDENCE OF ACCURACY ON THE STEP STRAIN AMPLITUDE

In Fig. 5, we demonstrate the degree of overlap between force chains obtained at different values of γ: starting from the same configuration (left column), we apply a step shear strain with amplitude γ (top row), and show the corresponding force networks (middle row) for a range of values of γ between $\gamma = 0.0$ and $\gamma = 0.16$. In the bottom row, we show the predictions made by a GNN trained only on force chains obtained at $\gamma = 0.02$. As γ increases, the prediction accuracy of the GNN decreases substantially – unlike a GNN conditioned on γ and trained on a data set containing multiple values of the step strain amplitude (see Fig. 5(b) of the main text).

FORCE CHAIN PREDICTION IN FRICTIONAL JAMMED GRANULAR SOLIDS

The frictional systems were simulated using the Discrete Element method (DEM) proposed by Cundall and Strack [10], which essentially models a granular material as a dense assembly of objects interacting through well-defined contacts. The latter are a combination of viscous and elastic elements of chosen strength. In this model, each contact consists of two springs (elastic component) and two dashpots (viscous component). One spring represents the restoring force due to overlap between parti-

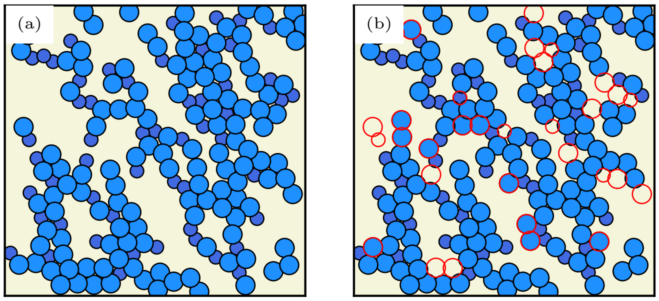

Supplementary Figure 6. (a) Force chains (blue) obtained for a configuration with $\mu = 1.0$ and $n_A = n_B = 200$, obtained through a shear simulation with step strain $\gamma = 0.05$. (b) Force chains predicted by a graph neural network taking as input the undeformed configuration in (a). Particles misidentified by the GNN are highlighted in red.

cles and another is associated with the tangential deformation of the contact; the latter is a term with a memory of the contact [10]. One of the two dashpots damps out the velocity along the radial direction and the other damps out the tangential velocity. For training data generation we performed simulations of two-dimensional frictional packings with frictional coefficients ranging from $\mu = 0.0$ (corresponding to the frictionless case) to $\mu = 1.0$. These simulations were performed using the LAMMPS package, which implements the DEM. We follow the protocol given in Silbert et. al. [11] to generate the frictional jammed packings. For this we first generate a dense frictionless packing by a sudden quench of configurations from $T = \infty$ to $T = 0.0$ and then relax the system to reach a force-free state. We then reduce the density by decompressing the system until we reach our desired area fraction $\phi \sim 0.95$. During this decompression and subsequent relaxation all the frictional parameters were switched on. We used $k_n = 2.0$, $k_t = 2.0$, $\gamma_n = 3.0$, $\gamma_t = \frac{1}{2}\gamma_n$ (see the original paper Ref. [10] for the definitions of these parameters) and a range of values for μ between 0.0 and 1.0. These parameters are chosen from a recent study on frictional jammed systems [12] that also employed the model by Cundall and Strack [10]. Following our previous protocol, these configurations (generated using different friction coefficients μ) are first deformed via a step strain ($\gamma \sim 0.05$), and we then identify the force chains using the same methodology as before. We train a GNN to predict, from the structure of the packing prior to the onset of shear deformation, which particles will end up being part of a force chain at the end of our simulation, in the same way as for the frictionless packings. We first trained a GNN on configurations obtained at $\mu = 1.0$, confirming that the GNN provides accurate predictions also for frictional particles (Fig. 6). Next, we trained a GNN conditioned on μ as described in the main text.

STRUCTURE FACTORS OF FORCE CHAINS PREDICTED BY GNN

As discussed in the main text, GNNs are able to predict force chains very accurately when applied to configurations with similar control parameters as observed during training, or when tasked to interpolate between its training regimes. However, when extrapolating to settings with very different control parameter values, the GNN prediction accuracy sharply drops. In this section (and Fig. 6 of the main text), we visualize the structural properties of the force chains the GNN predicts under different physical conditions, and also identify features the GNN misses out on when performing an extrapolation far from its training regime. We here consider the structure factor of the particles in the force chain

$$S(\mathbf{q}) = \frac{1}{N_c}\sum_{i=1}^{N_c}\sum_{j=1}^{N_c}\exp(-i\mathbf{q}(\mathbf{r}_i - \mathbf{r}_j)), \tag{5}$$

where N_c is the number of particles being part of a force chain. In Fig. 7, we show the structure factor of force chains predicted by two GNNs at two different area fractions; the GNNs were trained separately on the configurations for each value of the parameter. This demonstrates that the structure factor at $\phi = 0.86$ has strong anisotropic signatures whereas the one at $\phi = 1.0$ is close to isotropic – pointing towards a more branched structure at higher area fraction. Hence this is a clear example where the GNN prediction in an unknown system gives us very important information about the force chains under different, physically relevant conditions.

In Fig. 8, we show that the structure factor of the force chains for two different friction coefficients are almost identical, where the GNNs are trained at each of the frictional parameters. This is consistent with, and rationalizes, the fact that GNNs trained only on data with $\mu = 0.0$ are highly effective in predicting force chains for $\mu \neq 0.0$ (see Fig. 5(c) of the main text). It also underscores the relevance of the GNN in establishing the fact that at high enough area fractions, friction does not play a significant role. In Fig. 9, we show the structure factor of the force chains predicted by a GNN trained on $\gamma = \{0.02, 0.03, 0.04\}$ but applied to larger values of γ (see Fig. 5(b) of the main text), as well as the error w.r.t. the structure factor of the exact force chains. As the value of γ is increased further and further away from the training regime, the systematic structural bias in the GNN's prediction is revealed. The GNN predicts the force chains at higher values of γ to have a similar structure to the ones it observed during training at low values of γ. Hence, as γ increases – and the structure becomes more dissimilar to that seen during training – the bias in its predictions becomes more apparent. This is a clear

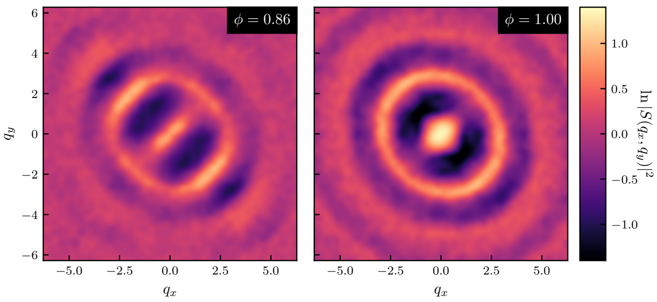

Supplementary Figure 7. Structure factor for particles predicted by a GNN to be part of a force chain in configurations obtained at $\phi = 0.86$ (left) and $\phi = 1.0$ (right). In the left panel, we used a GNN trained on $\phi = 0.86$ data, in the right panel we used a GNN trained on $\phi = 1.0$ data.

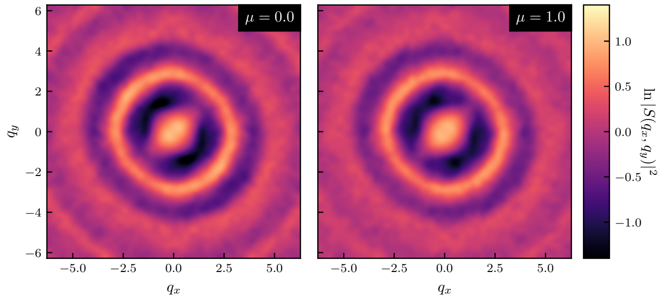

Supplementary Figure 8. Structure factor for particles predicted by a GNN to be part of a force chain in configurations obtained at $\mu = 0.0$ (left) and $\mu = 1.0$ (right). In the left panel, we used a GNN trained on $\mu = 0.0$ data, in the right panel we used a GNN trained on $\mu = 1.0$ data.

visualisation of the features that cause the accuracy of the GNN to drop significantly.

COMPARISON BETWEEN THE FORCE CHAIN LOCATIONS AND D^2_{min}

Previous machine learning studies on glassy systems have mainly focused on the calculation of 'softness', a structural predictor of regions susceptible to rearrangement [13–15]. It has been demonstrated quite extensively that softness is strongly correlated with D^2_{min} [16], which quantifies the magnitude of nonaffine displacement during a time interval Δt. For each particle i, it is calculated as

$$D^2_{\mathrm{min}}(i) = \min_{\Lambda} \frac{1}{|\mathcal{N}(i)|} \sum_{j \in \mathcal{N}(i)} (\mathbf{r}_{ij}(t + \Delta t) - \Lambda \mathbf{r}_{ij}(t))^2, \quad (6)$$

where the sum runs over the neighbours $\mathcal{N}(i)$ of particle i, $\mathbf{r}_{ij}$ is the displacement between particles i and j, $\Delta t \approx 9 \times 10^4$ and we minimize over the possible local strain tensors Λ. We also consider an instantaneous version of D^2_{min} [17]

$$D^{2'}_{\mathrm{min}}(i) = \min_{\Theta} \sum_{j \in \mathcal{N}(i)} [(\mathbf{v}_i - \mathbf{v}_j) - \Theta(\mathbf{r}_i - \mathbf{r}_j)]^2, \quad (7)$$

where the matrix Θ is the best-fit affine transformation matric, defined in a way analogous to Λ. $\mathbf{v}$ is the particle velocity defined as $\mathbf{v} = \mathbf{f}/\zeta$ where $\mathbf{f}$ is the force on a particle and ζ is the friction coefficient, and we again minimize over the possible local strain tensors. In Fig. 10 and Fig. 11, we demonstrate that our force chain prediction is not correlated to the local D^2_{min} (for both definitions) in the configuration prior to deformation. This indicates that our GNN has identified novel features in the structure of these configurations, which were not captured in earlier machine-learning based studies. Note that softness has been extensively used in various recent works [13–15] on connecting local dynamical response with structure through the use of machine-learning methods. Hence, here we aimed to address the natural question whether our GNN, which is successful in predicting properties of the force network, is picking up signals related to softness; our objective was not to establish any expected correlation (or absence thereof) between softness and force chains.

SUPPLEMENTARY REFERENCES

[1] T. Xie and J. C. Grossman, Phys. Rev. Lett. **120**, 145301 (2018).
[2] G. Li, C. Xiong, A. Thabet, and B. Ghanem, arXiv preprint arXiv:2006.07739 (2020).
[3] W. Hu, B. Liu, J. Gomes, M. Zitnik, P. Liang, V. Pande, and J. Leskovec, arXiv preprint arXiv:1905.12265 (2019).
[4] J. Gilmer, S. S. Schoenholz, P. F. Riley, O. Vinyals, and G. E. Dahl, in *International Conference on Machine Learning* (PMLR, 2017) pp. 1263–1272.
[5] M. Fey and J. E. Lenssen, in *ICLR Workshop on Representation Learning on Graphs and Manifolds* (2019).
[6] K. P. Murphy, *Machine learning: a probabilistic perspective* (MIT press, 2012).
[7] D. P. Kingma and J. Ba, arXiv preprint arXiv:1412.6980 (2014).
[8] J. F. Peters, M. Muthuswamy, J. Wibowo, and A. Tordesillas, Phys. Rev. E **72**, 041307 (2005).
[9] A. Tordesillas, D. M. Walker, and Q. Lin, Phys. Rev. E **81**, 011302 (2010).
[10] P. A. Cundall and O. D. L. Strack, Géotechnique **29**, 47 (1979), https://doi.org/10.1680/geot.1979.29.1.47.
[11] L. E. Silbert, Soft Matter **6**, 2918 (2010).
[12] H. Vinutha and S. Sastry, Nature Physics **12**, 578 (2016).
[13] E. D. Cubuk, S. S. Schoenholz, J. M. Rieser, B. D. Malone, J. Rottler, D. J. Durian, E. Kaxiras, and A. J. Liu, Phys. Rev. Lett. **114**, 108001 (2015).
[14] S. S. Schoenholz, E. D. Cubuk, D. M. Sussman, E. Kaxiras, and A. J. Liu, Nature Physics **12**, 469 (2016).
[15] J. W. Rocks, S. A. Ridout, and A. J. Liu, APL Materials **9**, 021107 (2021).
[16] M. L. Falk and J. S. Langer, Phys. Rev. E **57**, 7192 (1998).
[17] R. N. Chacko, P. Sollich, and S. M. Fielding, Phys. Rev. Lett. **123**, 108001 (2019).

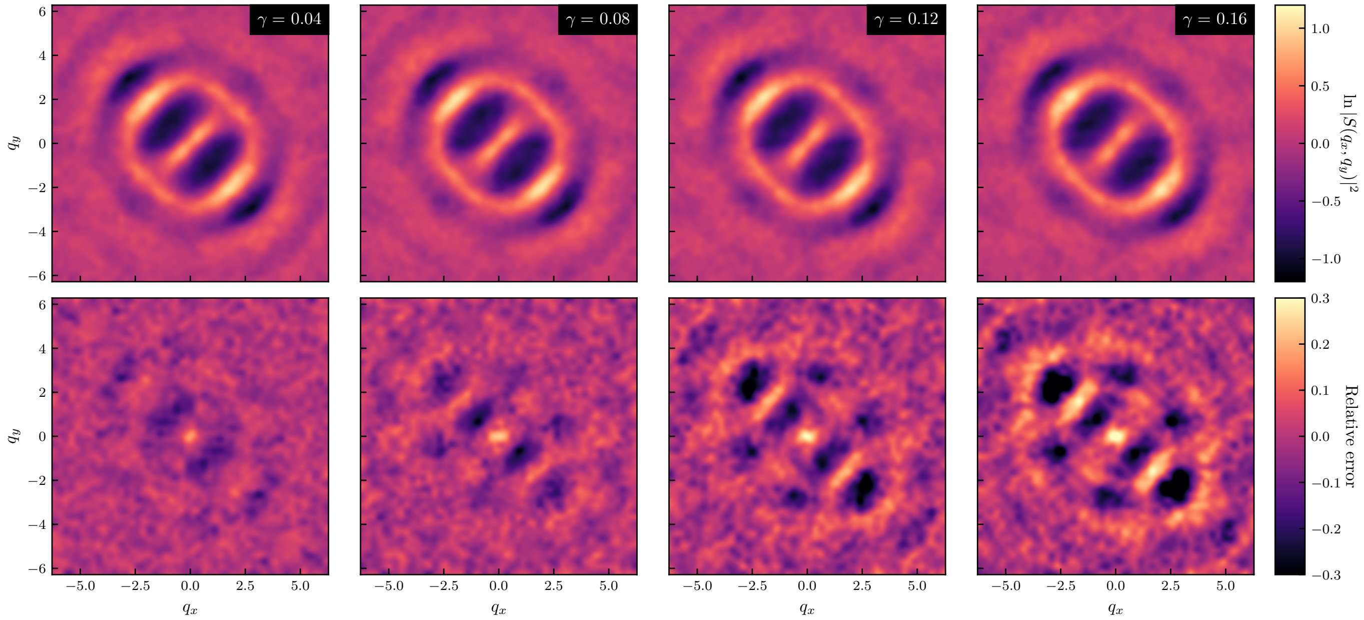

Supplementary Figure 9. Top row: Structure factor for particles predicted by a GNN to be part of a force chain for different values of γ. In all these cases, the GNN was only trained for $\gamma = 0.02, 0.03, 0.04$ (see Fig. 5(b) in the main text). Bottom row: the relative error compared to the exact values of the structure factor predicted by the GNN.

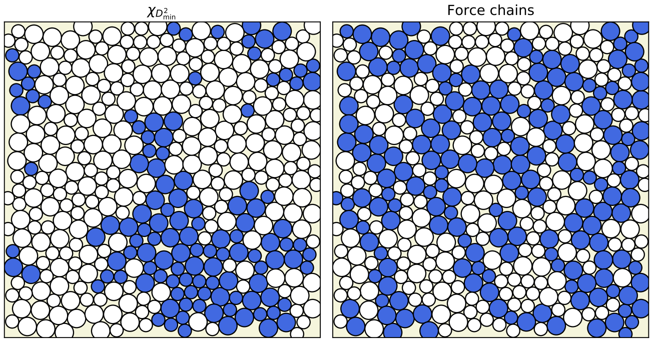

Supplementary Figure 10. Left: thresholded version $\chi_{D^2_{\text{min}}}$ of D^2_{min} (Eq. (6)): particles that had values of D^2_{min} in the top 30% before deformation are shown in blue. Right: Particles classified as being part of a force chain after deformation are shown in blue.

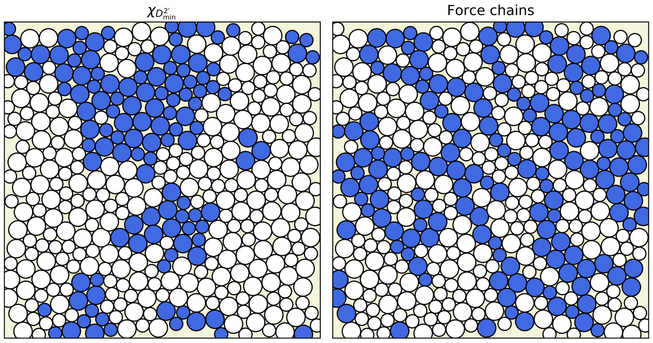

Supplementary Figure 11. Same as Fig. 10, but with the instantaneous version of D^2_{min}, as in Eq. (7).
